# Variable angle of insulated shotcrete under different loading rates and temperature and humidity cycles- shear test and analysis

**DOI:** 10.1371/journal.pone.0297381

**Published:** 2024-04-18

**Authors:** Gejun Tong, Jingzhao Shen, Jianyong Pang, Haotian Shi, Jinkun Huang

**Affiliations:** 1 State Key Laboratory for Safe Mining of Deep Coal and Environment Protection, Huainan Mining (Group) Co., Ltd, Huainan 232000, China; 2 School of Civil Engineering and Architecture, Anhui University of Science and Technology, Huainan, 232001, China; 3 China MCC17 Group Co., Ltd., No.88 Yu Shan Dong Road, Maanshan, Anhui Province, 243000, China; Shandong University of Technology, CHINA

## Abstract

The new thermal insulating shotcrete is of great significance for the management of thermal damage in deep mines, and its own strength has a greater impact on the roadway insulation and safe production, so it is very necessary to study the shear strength of the new thermal insulating shotcrete under the influence of the deep hot and humid environment and the stress of mining. For the heat-insulating shotcrete, firstly, we carried out the concrete variable angle shear test under different loading rates, which concluded that the shear rate and peak shear stress showed a trend of increasing and then decreasing; as the angle increases, the different rates have a greater impact on the peak shear stress of the specimen. Secondly, the concrete variable angle shear test was carried out under the temperature and humidity cycle, which revealed that the shear strength of thermal insulated shotcrete increased firstly and then decreased with the increase of temperature at the same number of cycles. Finally, the empirical equations between the cohesive force c, the angle of internal friction ϕ and the number of warm and wet cycles n and the temperature of warm and wet cycles T are fitted with the MATLAB software respectively, and the research results provide technical references for the management of geothermal temperature in deep well projects.

## 1. Introduction

The basic energy structure of China is characterized by "rich in coal, poor in oil, and low in gas", and coal plays an important role in China’s natural energy. It is predicted that by 2030, the total coal consumption in China will be 4.5~5.1 billion tonnes [1, 2]. With the large-scale mining and the gradual increase of energy demand in China over the years, deep-seated coal resources have gradually become the main target of mining. As the mining depth increases, the difficulty of mining also increases. Deep coal resources will encounter problems such as high ground stress, high ground temperature and high osmotic pressure, which will cause hidden dangers to safety production [3–5]. Among them, the effect of high ground temperature mainly causes mine heat damage, which is divided into two categories: high temperature heat damage and high humidity heat damage. According to the survey, there are 62 mines affected by mine heat damage in China, spreading over 13 provinces, among which the more prominent ones affected by mine heat damage are: the fifth mine of Pingcoal and the sixth mine of Pingcoal in Henan province, where the mining depth reaches -909m and -900m, respectively, and the surface temperature of the rock body in some areas is as high as 50°C and 53°C, and the underground hot water temperature reaches 42°C; the Dingji mine and the Panyi mine in Huainan, Anhui province, mining depths reached -826m and -810m, respectively, with local rock temperatures as high as 43°C and 40°C, and underground hot water temperatures of 45°C and reaching 35°C, respectively; Shenyang Hongyang third mine and Fushun Lao Hutai mine in northeast China, mining depths reached -1100m and -800m, respectively, with maximum rock surface temperatures of 50°C and 42°C, respectively, and maximum underground water temperatures of 51°C, respectively [6–9].

The field research shows that heat damage in mines can have a great impact on the physiology of underground workers. When the effective temperature of the working surface reaches 35°C, the human body temperature will be abnormal and sweating will increase sharply, which will lead to heat stroke or even death in serious cases. Long-term work of underground operators in a high-temperature environment can also lead to inability to concentrate, low work efficiency, and a significant increase in the incidence of safety accidents [10–12].

Numerous scholars have studied the problem of high-temperature heat damage in deep mines and developed corresponding control measures according to the heat source, and their measures are mainly divided into three categories: first, isolating the heat release from the surrounding rock, mainly laying insulation to isolate the heat energy transfer between the surrounding rock and the air; second, dissipating the heat in the air in the tunnel, mainly through circulating ventilation to reduce the temperature and optimizing the ventilation system; third, direct cooling methods of refrigeration systems, directly reducing the temperature in the tunnel through each refrigeration system, such as water cooling systems, mine air conditioning and thermoelectric cooling systems [13–15]. Relevant studies have shown that the exotherm from the surrounding rock accounts for nearly half of the total heat source, so isolating the exotherm from the surrounding rock is the most direct and effective method [16–18]. A new type of insulating hybrid fiber concrete was developed for the geothermal problem of deep mining. Insulated shotcrete is made by replacing a certain mass of stone sand with green vitrified amorphous glass beads in plain concrete and adding anticorrosive treated plant fibers to reduce the thermal conductivity of concrete [19–21]. Deep roadways are often affected by mining stress, mining stress is quasi-static load state, with the excavation project to produce disturbances, indoor test shear test using a change in the shear rate way to simulate quasi-static load [22]. In addition, the heat-insulating concrete in the underground roadway is attached to the surface of the roadway perimeter rock through the process of slurry spraying, and water sprinkling is used for maintenance, and the sprayed concrete is in the environment of the humid-heat cycle.

It can be seen that the deep stratum roadway perimeter rock heat-insulating concrete spray layer by the roadway dynamic pressure, high temperature and high humidity environment, the roadway perimeter rock is mainly shear damage [23–25]. Therefore, in-depth study of different loading rate and temperature and humidity cycle under the role of heat-insulating shotcrete shear resistance is very necessary. The article firstly carries out the formulation of heat-insulating concrete materials, and carries out the concrete variable angle shear test under consideration of different shear rates, and secondly carries out the shear test of heat-insulating concrete under temperature and humidity conditions, so as to analyse the adaptability of the heat-insulating concrete in the construction of deep mines, and the results of the research provide technical support for the management of high geothermal disasters in deep mining.

## 2. Test material and test preparation

### 2.1. Test materials

#### 2.1.1. Aggregate

**Fig 1:**
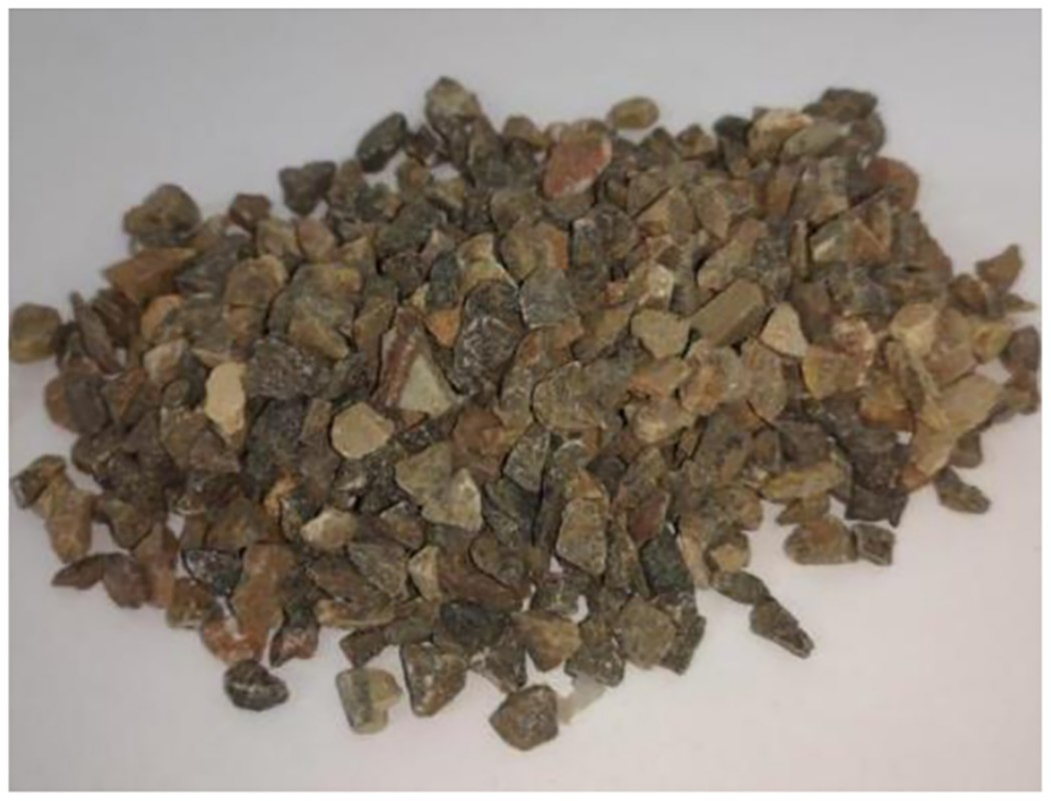
Limestone particles.

**Fig 2:**
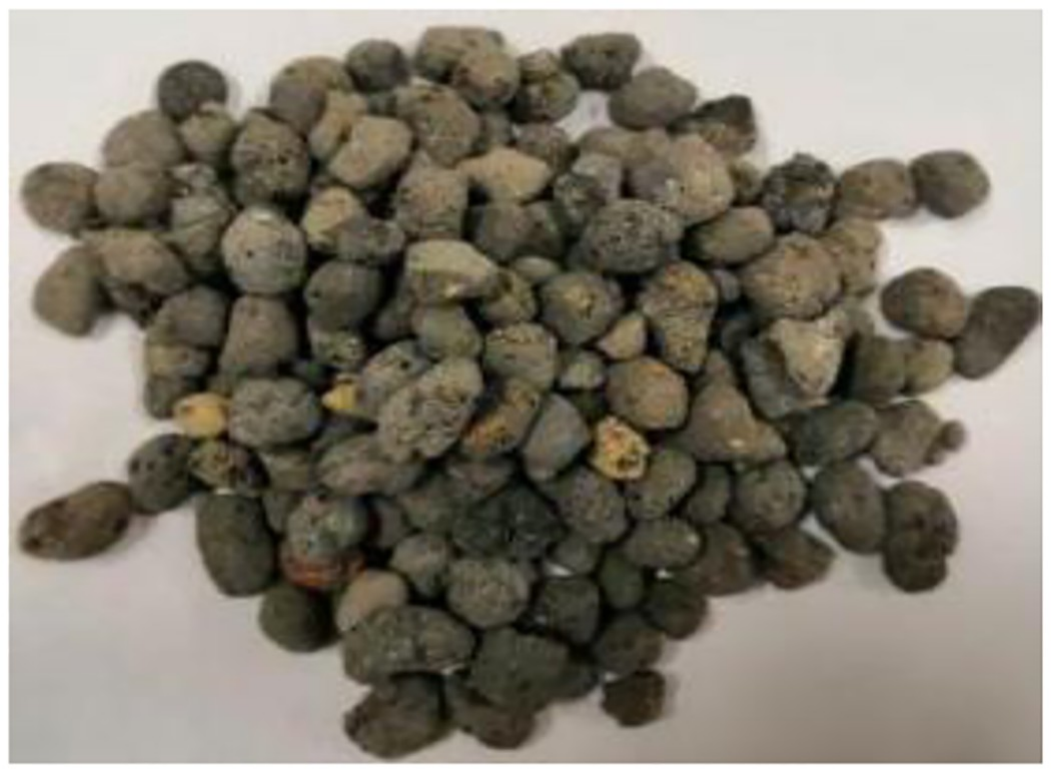
Ceramic granules.

**Fig 3:**
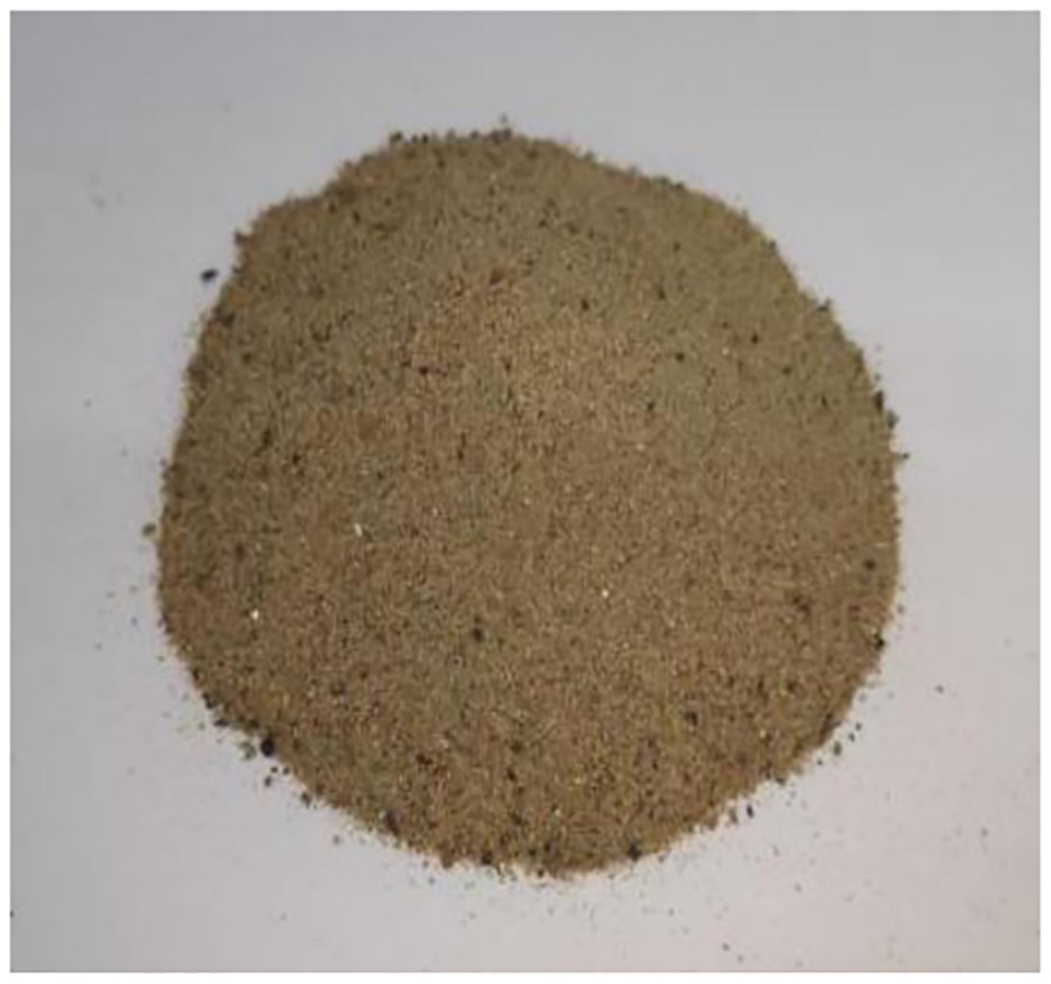
Sand.

**Fig 4:**
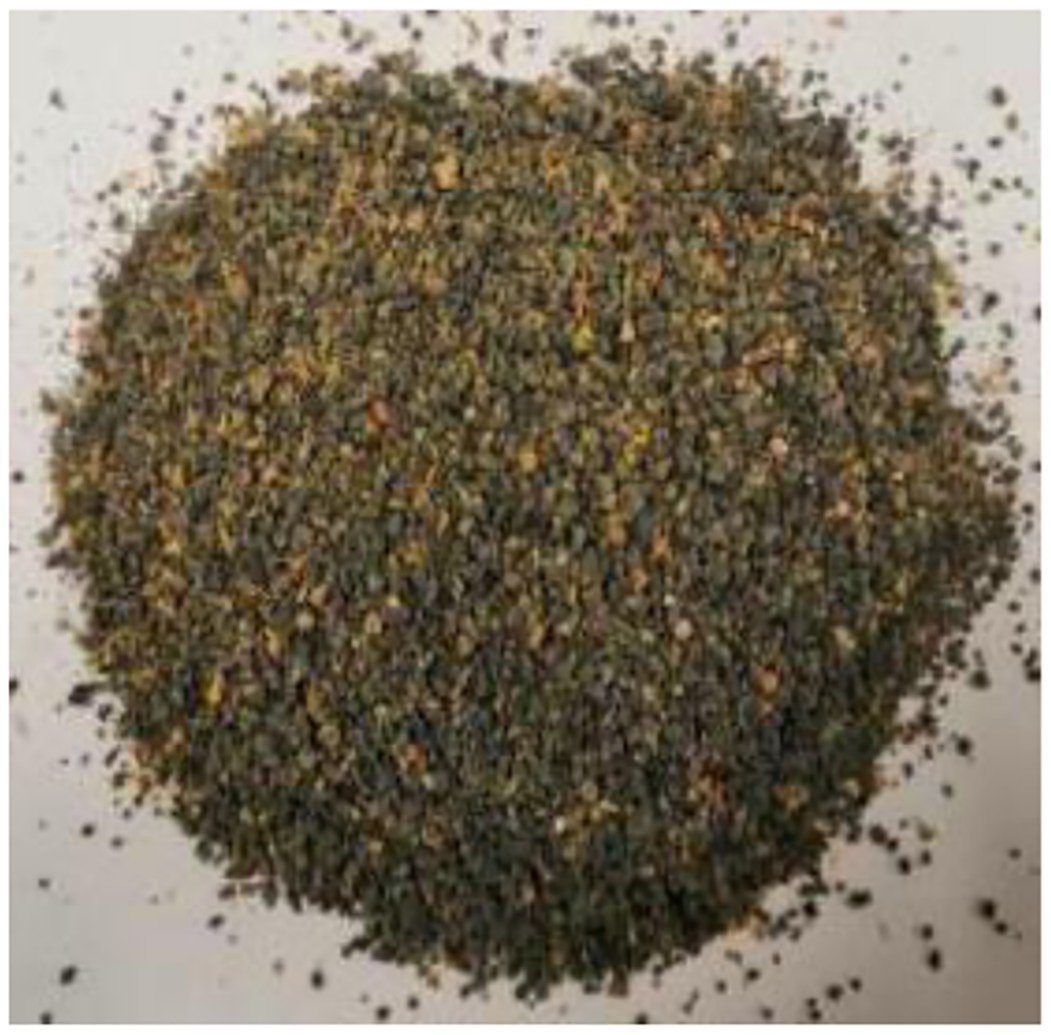
Ceramic sand.

**Fig 5:**
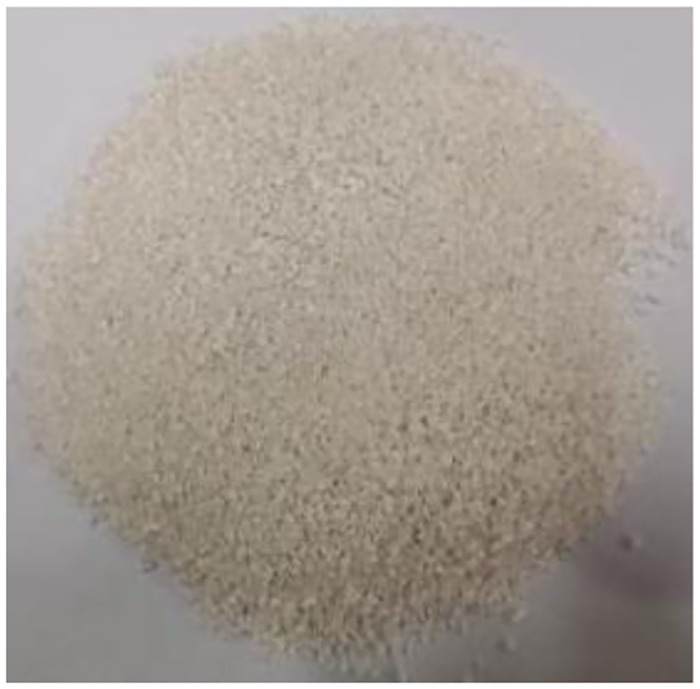
Glass beads.

- Stones
  The stone was selected from limestone particles according to the specification of "Technical Regulations for Shotcrete Application" (JGJT372-2016), as shown in Fig 1.
- Ceramic pellets
  Ceramic pellets are ceramic particles produced by foaming in a rotary kiln. Shale ceramic pellets were selected for the test. The selected ceramic granule is a smooth surface, such as the internal honeycomb coal-like spherical shape, as shown in Fig 2, and its main performance parameters are shown in Table 1.
- Sand
  The medium and coarse-grained river sand from Huainan was used in this test, as shown in Fig 3, whose particle size greater than 0.075 mm sand accounted for more than 80% of the total, and the fineness modulus was measured by sieving as 2.8, which satisfied the requirements of the sand needed for the test. In order to reduce the test error, the sand needs to be cleaned to reduce the mud content; the sand needs to be sieved to remove stones and mud lumps; the sand needs to be dried to prevent moisture and reduce the moisture content. After treatment, the measured sand mud content was 1.4% and light matter content was 0.1% [26].
- Ceramic sand
  The general particle size of pottery granules is between 5 and 20 mm, while the particle size lower than 5 mm is called pottery sand, as shown in Fig 4. Both pottery sand and pottery granules have high strength, corrosion resistance, earthquake resistance, low thermal conductivity and other properties, while the density of pottery sand is slightly higher than that of pottery granules, and its performance parameters are shown in Table 2 [27].
- Glass beads
  Glassy microbeads are an inorganic glassy mineral material made by multi-stage silicon carbide electric heating tube type production technology. Water-repellent glass beads are chosen for the test, as shown in Fig 5, and their main physical properties are shown in Table 3.

**Table 1:** Performance parameters of ceramsite.

Bulk density /g·cm^-3^	Distribution of grain size /mm	Porosity /%	compressive strength of concrete cylinder /MPa	Thermal conductivity /W·(K·m)^-1^
0.56	≤15	40	4.0	≤0.52

**Table 2:** Performance parameters of ceramic sand.

Bulk density /g/cm3	Particle grading /mm	Porosity /%	Compressive strength of concrete cylinder /MPa	Thermal conductivity /W·(K·m)-1
0.6	≤5	37	5.0	≤0.45

**Table 3:** Physical properties of glass beads.

Grain size /mm	Density /g·cm^-3^	Thermal conductivity /W·(K·m)^-1^	Water absorption /%	Closed porosity /%	Volume loss ratio /%	Service temperature /°C
0.15~0.5	0.1	0.036	30	90	42	800

#### 2.1.2. Gelling material

- Cement
  Shotcrete requires cement with high early strength, short initial setting time, and good homogeneity. The cement selected for this test is P·O42.5 ordinary silicate cement.
- Fly ash
  Fly ash replaces part of the cement, not only can reduce the cost, but also can reduce the heat of hydration of concrete, reduce the permeability performance, and improve the durability of concrete. This test uses Class I fly ash.

#### 2.1.3. Fiber material

**Fig 6:**
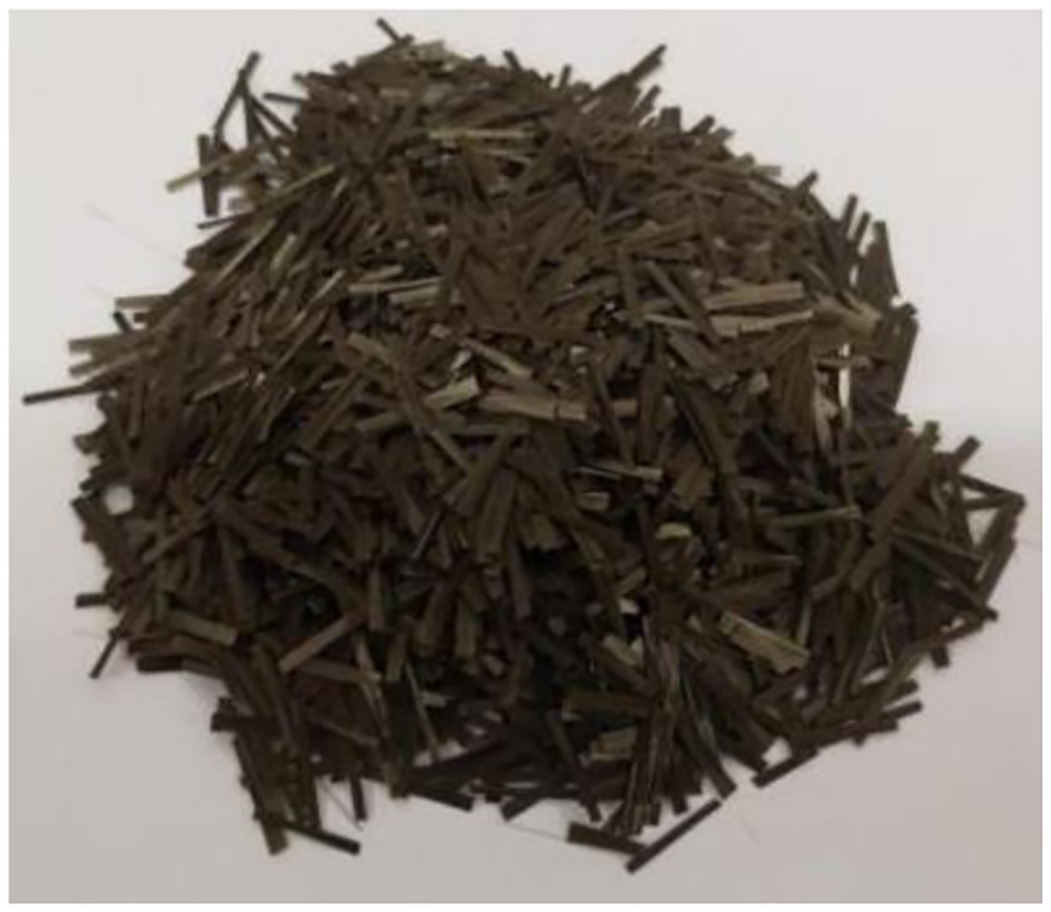
Basalt fiber.

**Fig 7:**
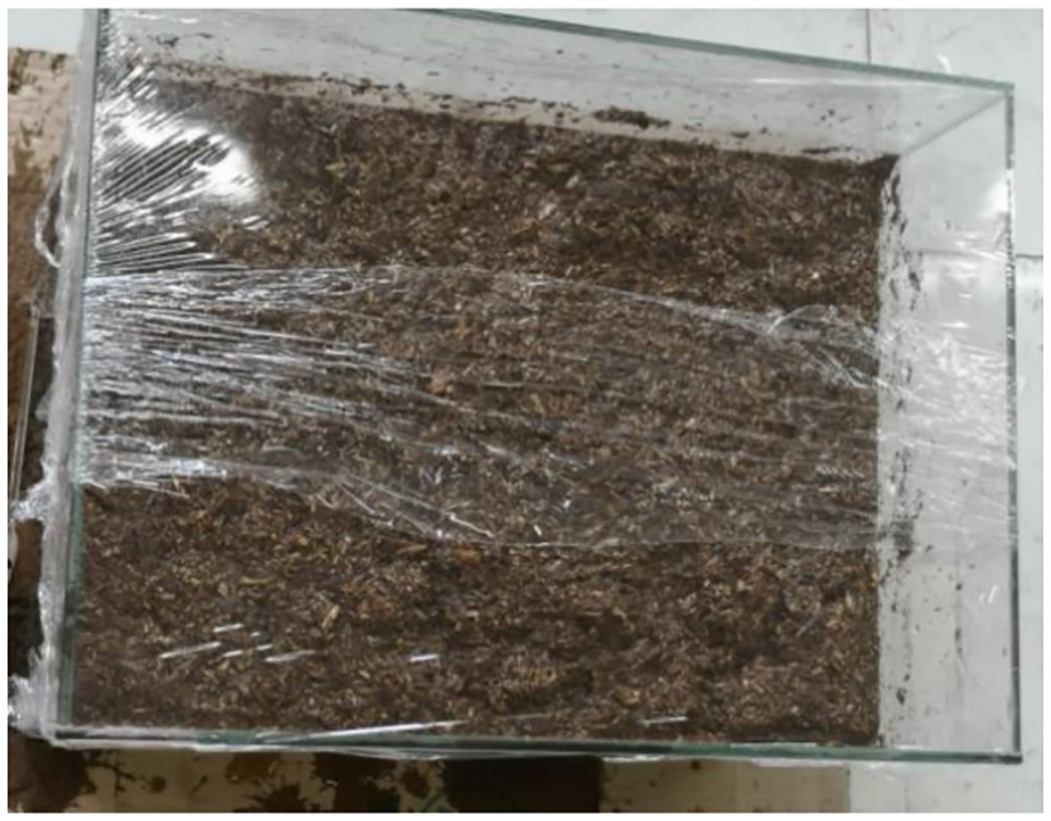
Straw fiber.

- Basalt fiber
  Basalt Fiber (BF) is known as a pollution-free "green industrial material" in the 21st century as shown in Fig 6. It is a soft fiber made from basalt in a furnace at 1450–1500°C after thousands of refinements, which has low thermal conductivity, high temperature resistance, low temperature resistance, corrosion resistance, flame retardancy, high tensile strength, high elastic modulus, etc. The specific mechanical properties are shown in Table 4, corrosion resistance, flame retardant, high tensile strength, high modulus of elasticity, and other excellent properties, and the specific mechanical properties are shown in Table 4 [28, 29].
- Straw fiber
  Recent studies have shown that straw has good thermal conductivity, hard surface, good toughness, greater tensile strength and other excellent properties. Straw fiber can be added to the concrete, and the concrete structure is better combined, when the concrete damage, straw fiber can hinder the splash of concrete debris. This test choose polyvinyl alcohol pva aqueous solution to straw fibers for anti-corrosion treatment, after treatment as shown in Fig 7 [30–32].

**Table 4:** Mechanical properties of basalt fibers.

Mechanical	Density /g·cm^-3^	Elastic modulus /GPa	Tensile strength /MPa	Elongation at break /%
Basalt fiber	2.6~2.8	79.3~93.1	3000~4840	3.2

### 2.2. Specimen preparation

According to the research results of the group, the optimal mixture of ceramic granule, sand, basalt fiber and straw fiber was selected by orthogonal test, and the test ratio is shown in Table 5.

**Table 5:** Mix proportion of thermal insulation shotcrete.

Sand /Kg	Ceramic sand /Kg	Glass beads /Kg	Pebble /Kg	Ceramic aggregate /Kg	Straw fiber /Kg	Basalt fiber /Kg	Cement /Kg	Fly ash /Kg	Water /Kg	Water reducing admixture /Kg
764.152	66.448	9	772.458	58.142	0.4	5.26	427.5	47.5	213.8	3.8

**Fig 8:**
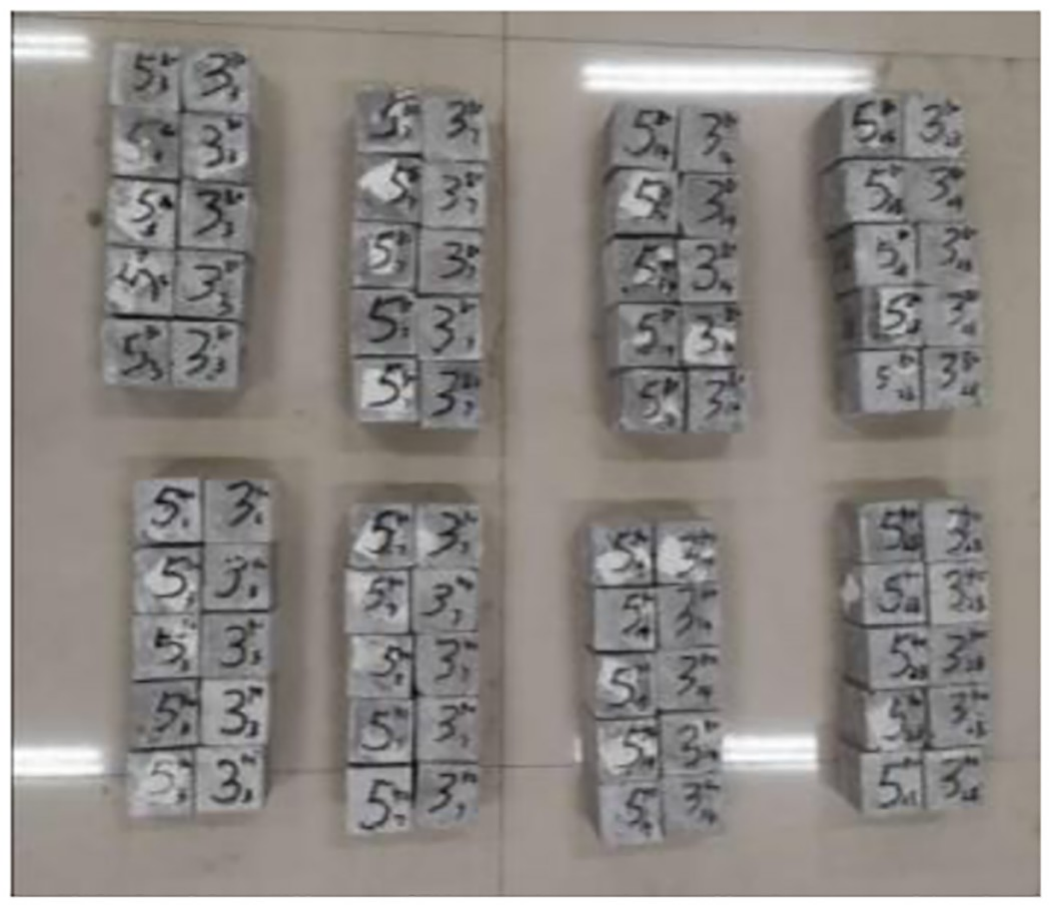
Test specimen (28 days of maintenance).

Concrete variable angle shear performance tests were performed according to the concrete specification by making 50 mm × 50 mm × 50 mm cubic test blocks [33–35]. The amount of materials was first calculated according to the concrete mix ratio, and the raw materials were weighed at 1.2 times the calculated amount considering the loss in the concrete making process. Sequentially add stone, sand, ceramic granule, amalgamated sand in the mixer, turn on the mixer and mix for 30 s. Then add cement, fly ash, mix for 30 s, add basalt fibre, straw fibre, mix for 60 s. Then add water, water reducing agent, mix for 60 s. Unload the concrete material, load it into the standard touching tool, cover it with cling film, and wait for the specimen to be shaped, then dismantle the touching tool, put the specimen into the saturated GaOH solution for 28 days, and some specimens are shown in Fig 8.

## 3. Test equipment and test program

Shotcrete in deep ground engineering is prone to shear damage, and is affected by deep roadway or coal mining face mining, the existence of dynamic pressure influence on the roadway surrounding rock and deep strata high temperature and high humidity environment. Therefore, the first to consider the impact of different shear rate under the variable angle shear test, followed by the temperature and humidity cycle under the effect of variable angle shear test. Specifically as follows.

### 3.1. Concrete variable angle shear test under different loading rates

**Fig 9:**
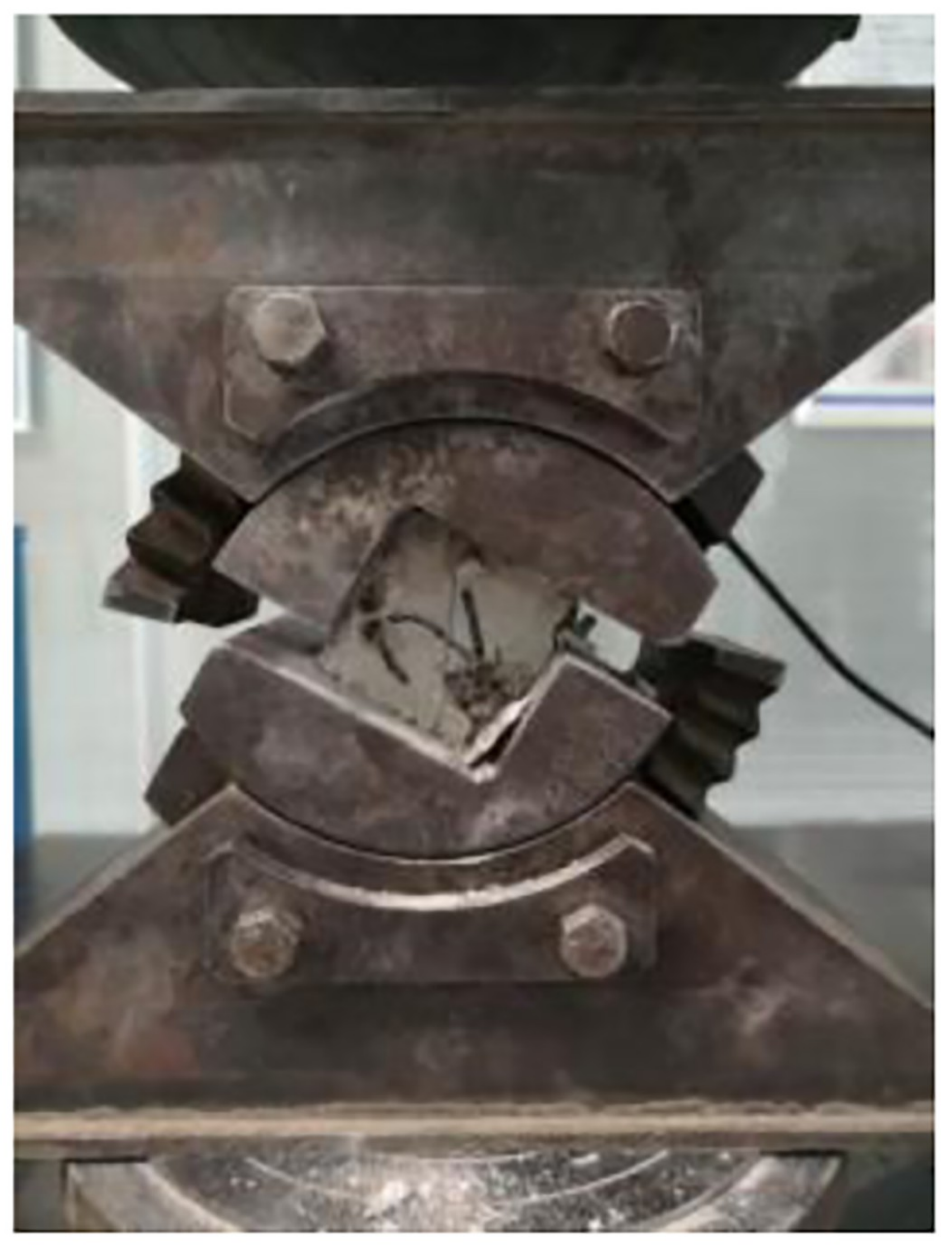
Variable angle shear jig.

**Fig 10:**
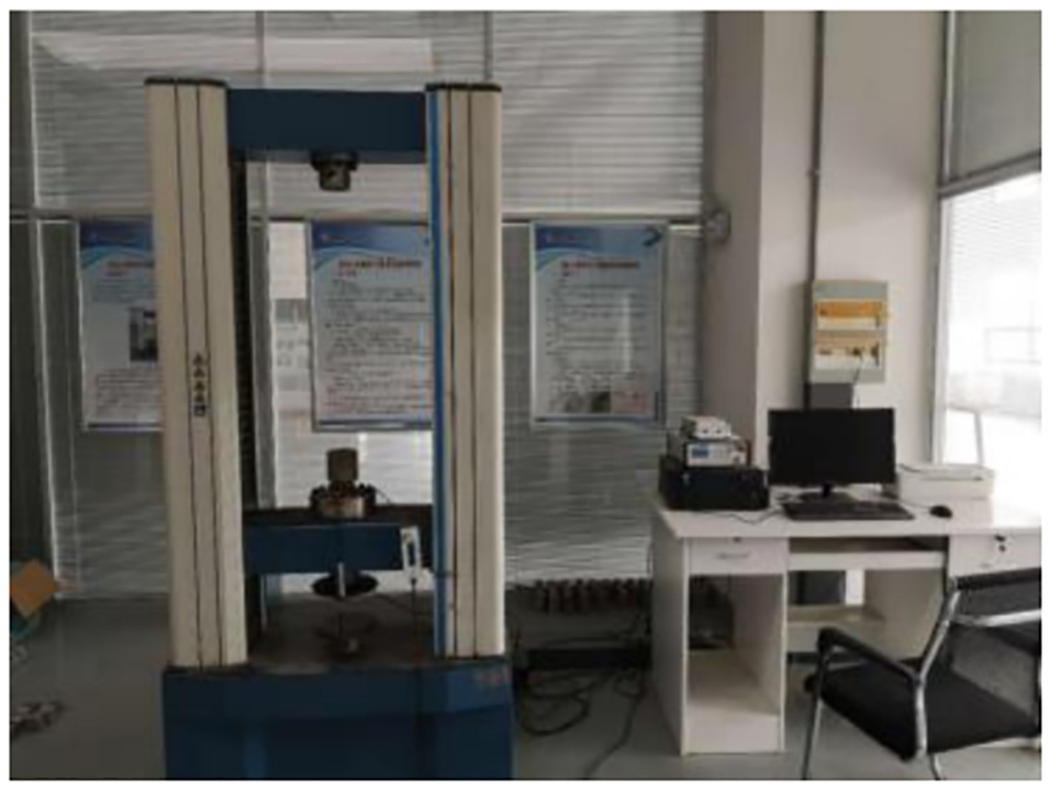
RDL testing machine.

The variable angle shear test was carried out on the high-temperature creep endurance strength testing machine (RDL) using the variable angle shear jig, as shown in Figs 9 and 10. the dimensions of the RDL tester were 710×550×2210 mm. the test blocks were divided into groups of three, and 20 groups of test blocks were taken and set at five angles of 30°, 38°, 46°, 54°, and 62° using the variable angle shear jig, and each angle was set at four loading rates of 1 mm/s, 3 mm/s, 5 mm/s, and 7 mm/s, respectively. mm/s, 5 mm/s, 7 mm/s, and shear a group of test blocks at each loading rate.

### 3.2. Concrete variable angle shear test under temperature and humidity cycles

In order to test the strength change of insulated concrete in a high temperature and high humidity tunnel, the test blocks were subjected to high temperature and high humidity to simulate the environmental effects of concrete in the tunnel. 15 groups of 9 test blocks each were selected for temperature and humidity cycling tests.

**Fig 11:**
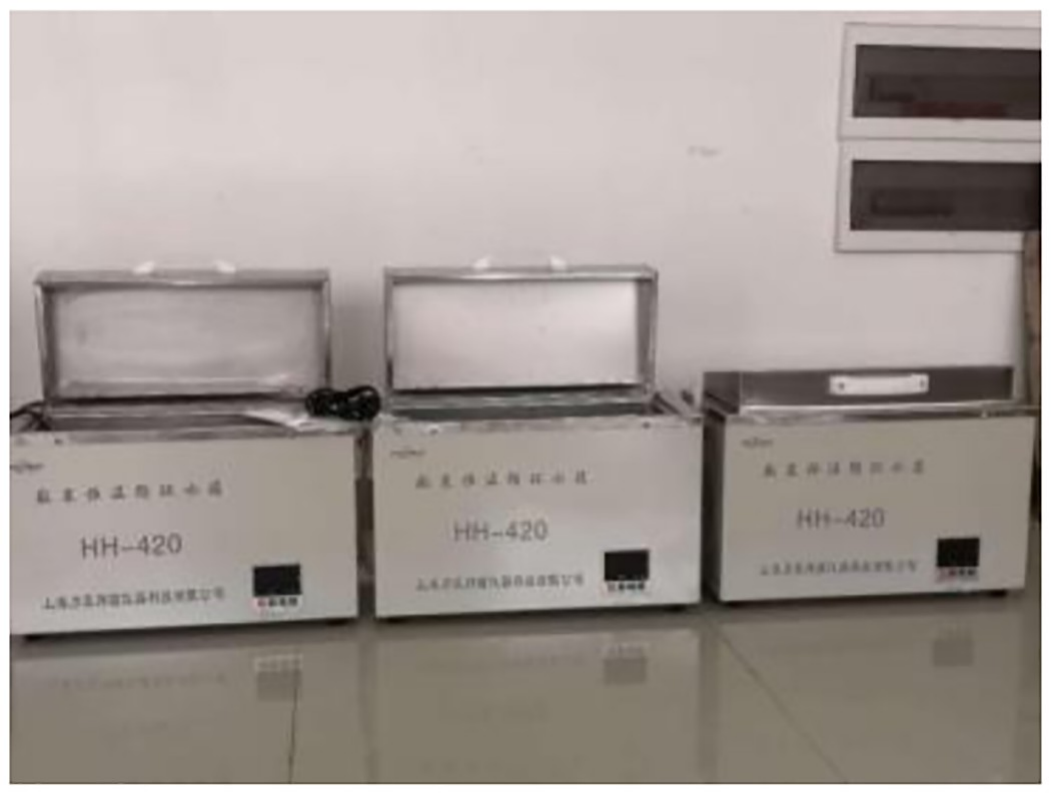
HH-420 thermostatic water bath box.

**Fig 12:**
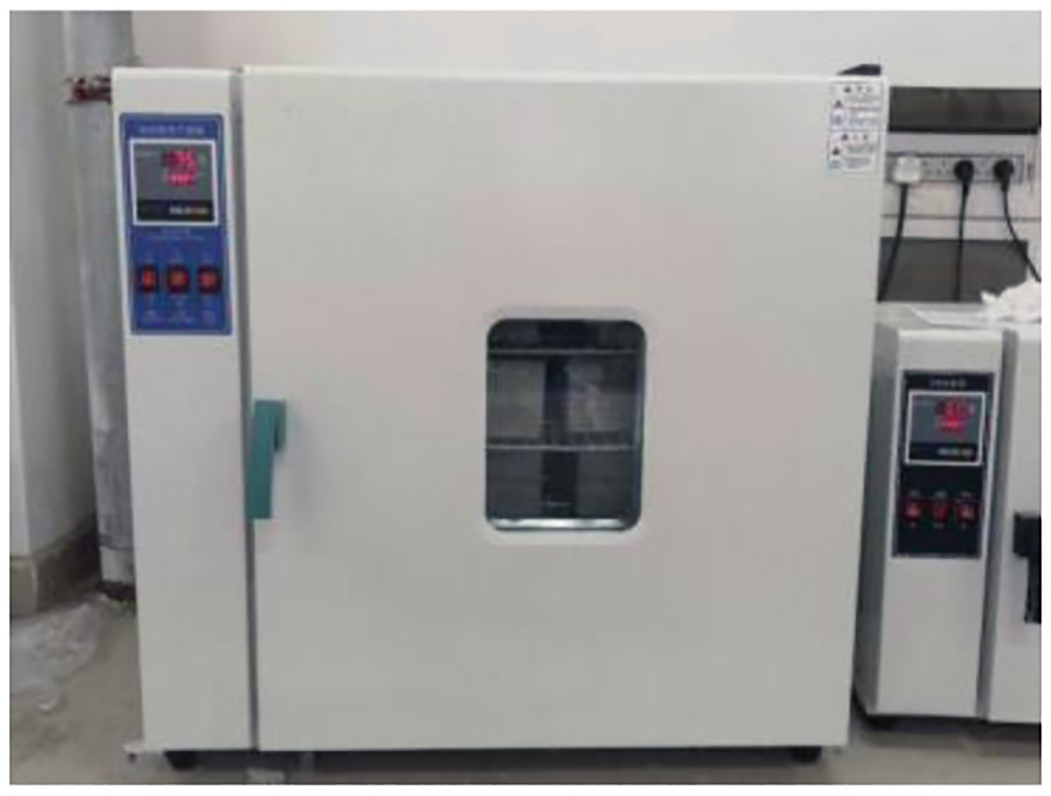
XMA-2000 drying box.

The specific steps of the primary temperature and humidity cycle are as follows:

Use three HH-420 thermostatic water baths to heat the groundwater to 20°C, 40°C, 80°C, as shown in Fig 11, test blocks were placed in the three water baths, immersed in 16h. Take the test block out of the water bath and put it into XMA-2000 type drying oven with heating set at 60°C for drying, as shown in Fig 12, drying for 6h. After drying, remove the test block and let it stand for 30min.

15 groups of test blocks were subjected to 3, 7, 14, 28 and 56 cycles of temperature and humidity in water at 20°C, 40°C and 80°C respectively. At the end of 3 cycles, the test blocks were sealed with cling film to avoid contact between the test blocks and air, and the other cycles were continued, and so on for 7 cycles, the test blocks were sealed with cling film, and the other test blocks were continued, and so on for 56 cycles, and so on for different cycles. All test blocks together for 46°, 54°, 62° three angles of variable angle shear.

## 4. Test results and analysis

### 4.1. Results and analysis of concrete variable angle shear tests at different rates

#### 4.1.1. Specimen shear stress and positive stress relationship

**Fig 13:**
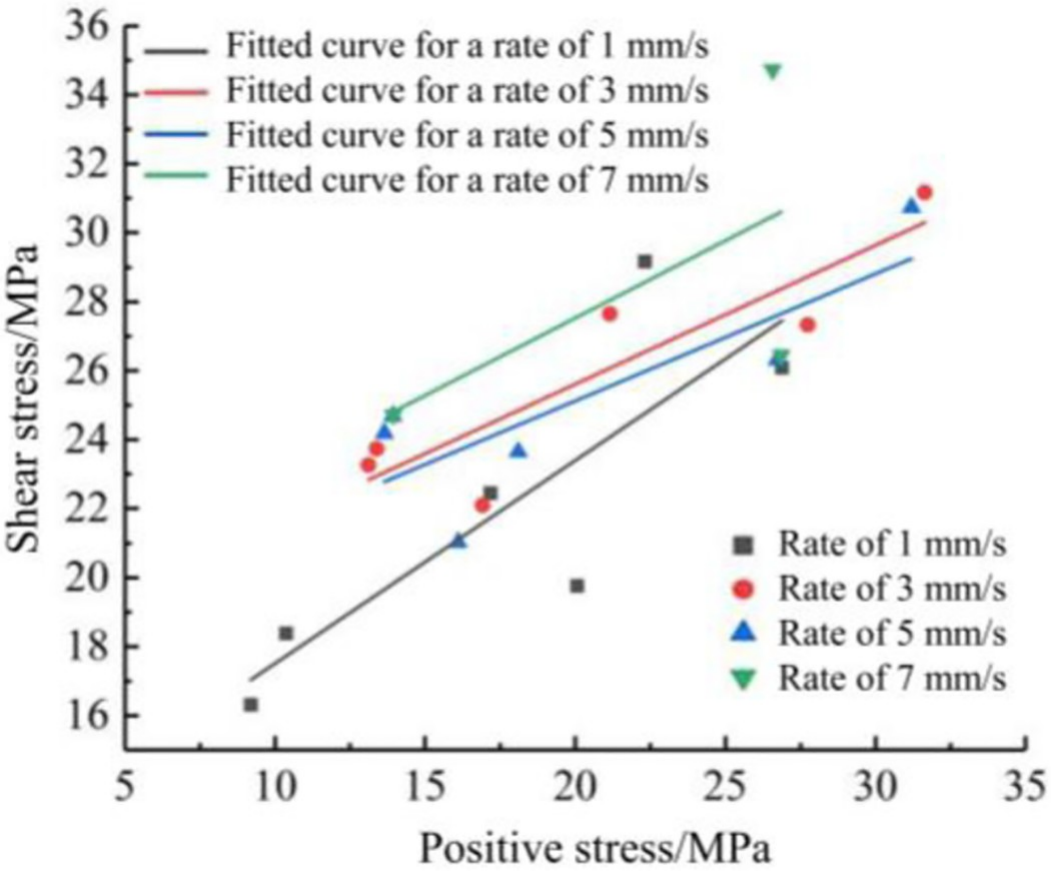
Different rates vs. peak shear stress curves.

The linear fits were taken to 46°, 54°, and 62° for linear fitting, as shown in Fig 13. The cohesive force of shotcrete at a rate of 1 mm/s is 11.62 MPa and the internal friction angle is 30.49°, at rates of 3 mm/s and 5 mm/s the cohesive force is 17.55 MPa and 17.75 MPa, respectively, and the internal friction angle is 21.94° and 20.23°, respectively, and at a rate of 7 mm/s the cohesive force is 18.55 MPa and the internal friction angle The internal friction angle was 24.17°. The internal friction angle decreases and increases at different rates, with the lowest angle of 20.23° at 5 mm/s and the highest angle of 30.49° at 1 mm/s. As the loading rate increases, the cohesive force also increases gradually, and the cohesive force is proportional to the loading rate. The internal friction angle decreases and then increases as the rate increases.

#### 4.1.2. Effect on peak shear strength at different loading rates

**Fig 14:**
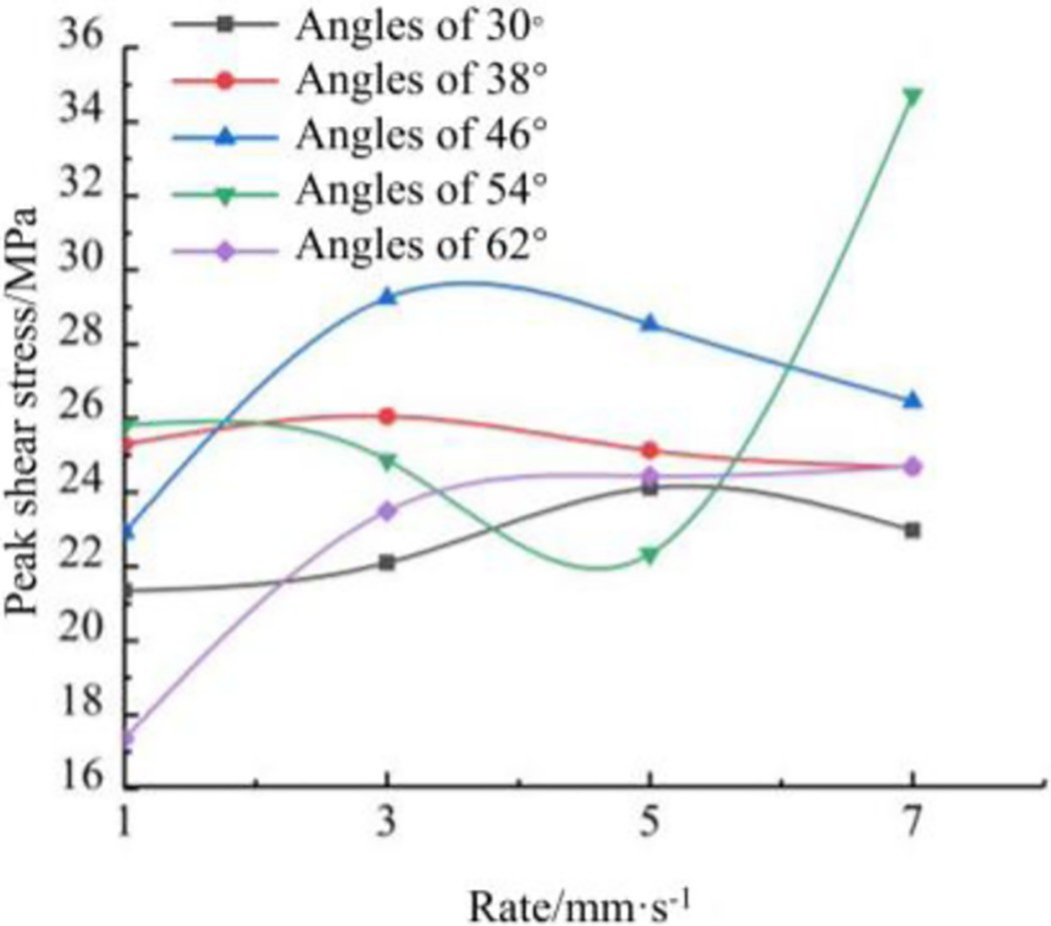
Specimen shear stress curves vs. positive stress curve.

As shown in Fig 14, the peak shear strength at a shear angle of 30° was 21.34 MPa at a rate of 1 mm/s. When the rate was increased to 3 mm/s, the shear strength increased by 3.5% to 22.1 MPa, and when the rate was increased to 5 mm/s, the shear strength continued to increase by 13% to 24.12 MPa, and when the rate was increased to 7 mm/s, the shear strength began to The shear strength decreased to 22.97 at 7 mm/s, which was 4.8% lower than the rate of 5 mm/s and 7.6% higher than the rate of 1 mm/s. The shear strength of shear angle 38° and 46° at different rates had the same trend of increase and decrease, and the shear strength of angle 46° was more affected by different rates, and the shear strength of angle 38° was less affected by different rates, and the shear strength changed smoothly, and the peak shear strengths were 25.29 MPa and 22.92 MPa at the rate of 1 mm/s, respectively. The shear strength of shear angle 38° and 46° decreased continuously when the rate was increased to 5 mm/s and 7 mm/s. The shear strength decreased by 5.3% and 9.6% at the rate of 7 mm/s compared with the rate of 5 mm/s, respectively. At a shear angle of 54°, the rates of 1 mm/s, 3 mm/s and 5 mm/s showed a decreasing trend, and the rate of 5 mm/s decreased by 13.44% compared with the rate of 1 mm/s. The rate increased to 7 mm/s, which increased by 55.42%. At a shear angle of 62°, the shear strength increased as the rate increased, from 17.35 MPa to 24.71 MPa from rate 1 mm/s to rate 7 mm/s, an increase of 42.42%. As the shear angle becomes larger, the shotcrete is subjected to shear stresses along with the effect of tensile stresses and changes in positive stresses leading to changes in cohesion, so different angles are affected by different trends in the rate. It can be seen that as the rate increases the shear strength first increases and then decreases trend. As the angle increases, the greater the effect of different rates on the peak shear stress of the specimen, the smaller the shear angle, the lower the effect of different rates, the smoother the curve. The larger the shear angle, the greater the effect of different rates on the shear stress, the peak shear stress curve is relatively jumpy.

#### 4.1.3. Characteristics of shotcrete damage under shear stress

**Fig 15:**
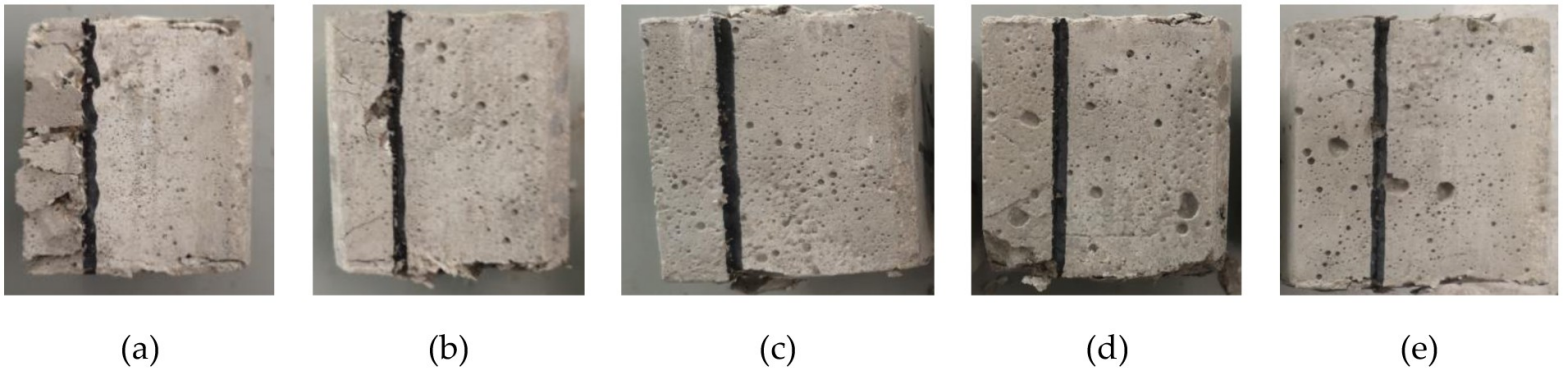
Shear damage form under different angles: (a) Shear angle 30°; (b) Shear angle 38°; (c) Shear angle 46°; (d) Shear angle 54°; (e) Shear angle 62°.

According to the analysis of the data in Table 1, it is found that at angles 30° and 38°, the shear surface is damaged by both positive and shear stresses because the positive stress is much greater than the shear stress, and the positive and shear stresses do not conform to the Coulomb strength criterion. Fig 7 gives the shear damage forms of the specimens at angles 30°, 38°, 46°, 54° and 62° at a rate of 1 mm/s. Fig 15(a) shows that the positive stress of 39.18 MPa is much greater than the shear stress of 21.34 MPa at the shear angle of 30°, and part of the concrete on the left side of the shear damage surface is crushed to show a broken form. Fig 15(b) can be seen in the shear angle of 38°, the positive stress than the angle of 30° lower 5.09 MPa, shear damage to the left side of the concrete cracks and was not destroyed. Fig 15(c)–15(e) gives the angle of 46°, 54°, 62° when the specimen shear damage form, these three angles of concrete completely in accordance with the shear angle line damage, the surrounding concrete intact without cracks or broken situation. The specimen must be completely destroyed by shear strength under the positive stress and shear stress to comply with the Cullen strength criterion.

**Fig 16:**
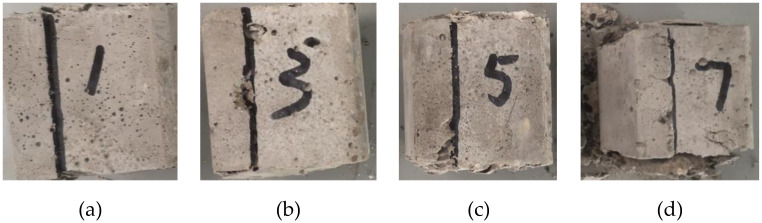
Damage form of specimen bottom surface at different rates: (a) Loading rate 1 mm/s; (b) Loading rate 3 mm/s; (c) Loading rate 5 mm/s; (d) Loading rate 7 mm/s.

Fig 16 shows the damage of the specimen at shear angle 46° at loading rates of 1 mm/s, 3 mm/s, 5 mm/s and 7 mm/s. At the loading rate of 1 mm/s, only a few cracks appear on the front side of the specimen, and the bottom side is completely damaged by the shear angle line, with the surrounding concrete intact. At a loading rate of 3 mm/s, the specimen showed obvious cracks on the front side and the bottom side was still intact. At a loading rate of 5 mm/s, a large piece of concrete falls off from the front side of the specimen, and the concrete at the bottom left corner is damaged. At a loading rate of 7 mm/s, the specimen was broken into several pieces and cracks could be seen along the shear surface around the bottom. As the loading rate increased, the degree of specimen fragmentation gradually deepened.

### 4.2. Results and analysis of concrete variable angle shear test under temperature and humidity cycles

#### 4.2.1. Analysis of the effect of different cycle times on the shear strength of concrete

The shear properties of concrete under the action of 40°C temperature and humidity cycles were analyzed at angles 46°, 54° and 62°. The shear stress curves of concrete under the action of different cycles shown in Fig 16 were obtained.

**Fig 17:**
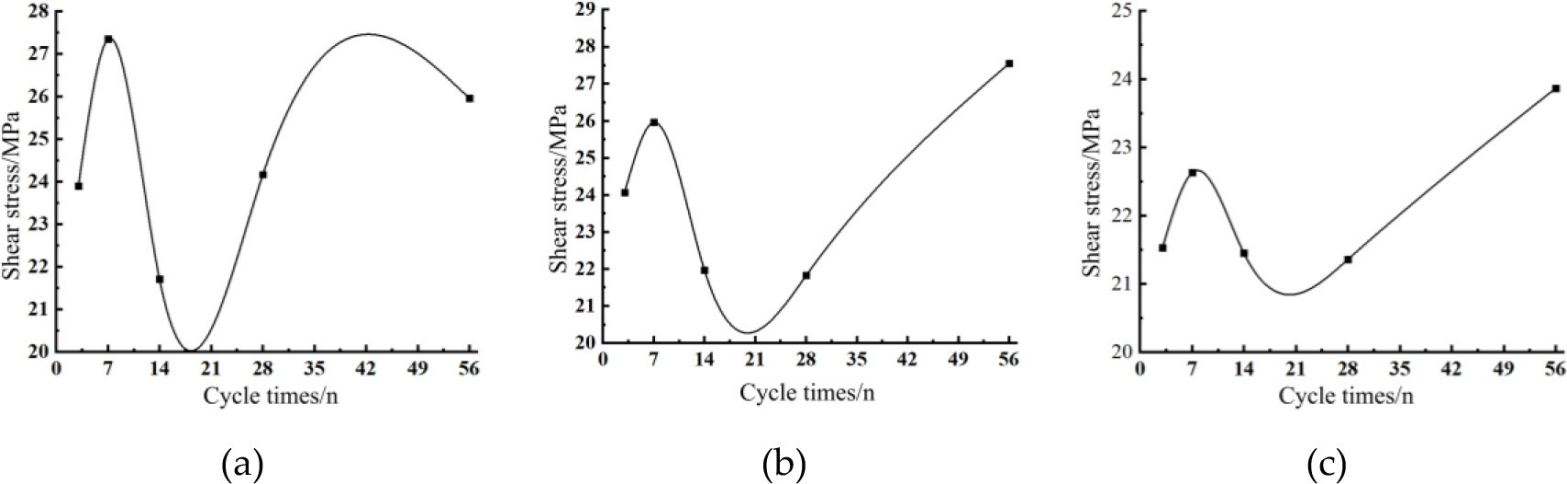
Different shear angle shear stress-cycle number relationship curve: (a) Shear angle 46°; (b) Shear angle 54°; (c) Shear angle 62°.

As can be seen from Fig 17(a), the shear angle of 46°, with the increase in the number of cycles, the shear strength of concrete changes M-shaped, cycle 3 times to 7 times, the shear strength increased, rising 14.40%, the peak shear strength at cycle 7; cycle 7 times to 18 times, the shear strength decreased significantly, down 26.87%; cycle 14, 28 times, 56 times, the shear strength increased respectively 11.33%, 7.40%, from the curve, the shear strength increased first and then decreased process from 28 to 56 cycles.

Fig 17(b) shows, the shear strength of concrete changes with the increase of the number of cycles at the shear angle of 54° in an N-shape, the shear strength increases from 3 cycles to 7 cycles and rises by 7.85%; the shear strength decreases from 7 cycles to 20 cycles and falls by 21.42%, and the shear strength continues to increase from 20 cycles to 56 cycles.

From Fig 17(c), the shear strength of concrete changes with the increase in the number of cycles at a shear angle of 62° in an N-shape, with 3 cycles to 7 cycles, the shear strength increases and increases by 5.11%; with 7 cycles to 20 cycles, the shear strength decreases and decreases by 8.09%; with 20 cycles to 56 cycles, the shear strength continues to increase.

In summary, the concrete strength tends to increase then decrease and then continue to increase under the effect of 40°C temperature and humidity cycles. The concrete reached the peak shear strength at 7 cycles of temperature and humidity, and the highest shear strength increase was observed at a shear angle of 46°. At 20 cycles of temperature and humidity, the concrete had the lowest shear strength and the highest decrease in shear strength at a shear angle of 46°.

#### 4.2.2. Analysis of the effect of different cyclic temperatures on the shear strength

**Fig 18:**
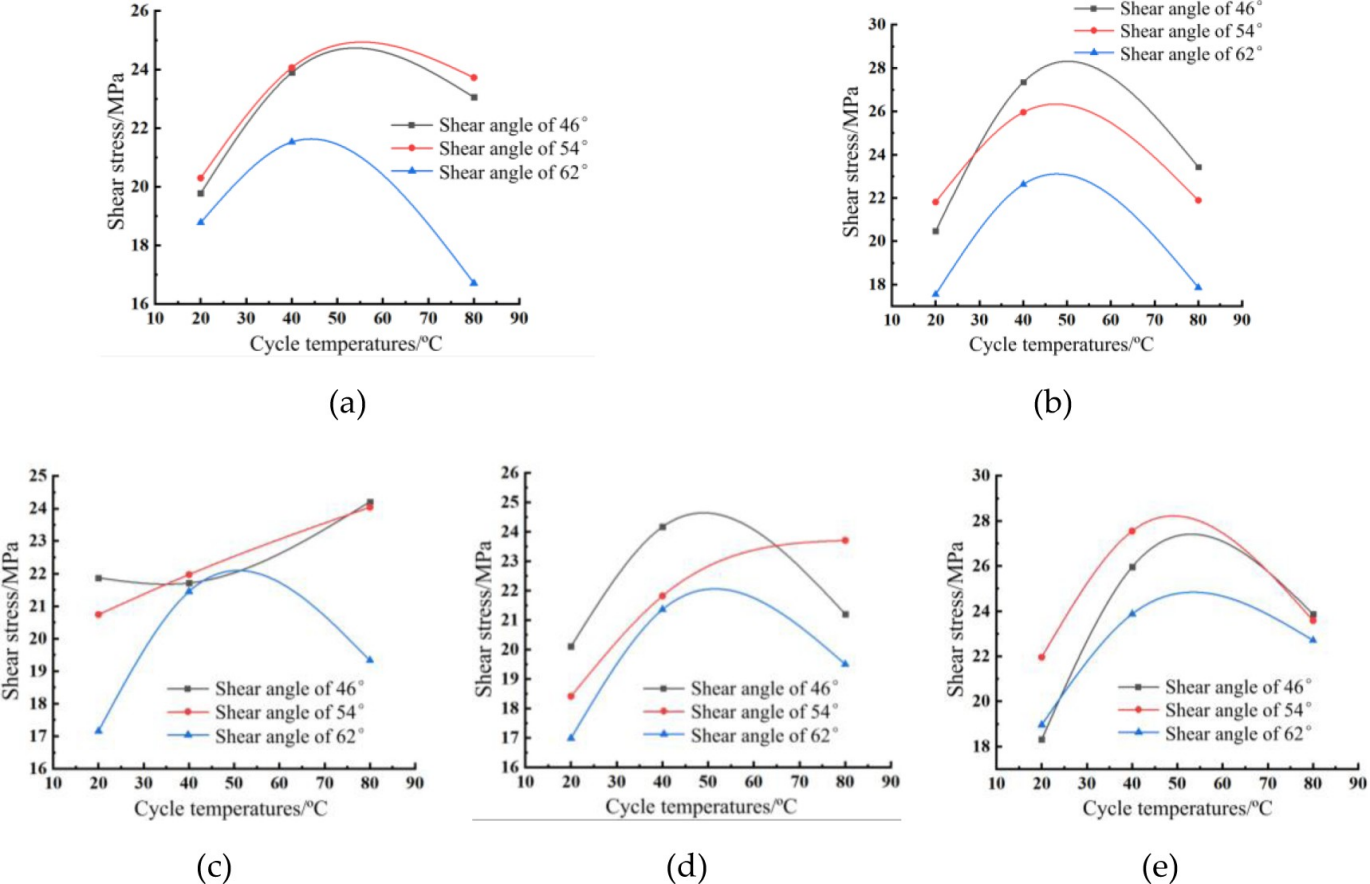
Cyclic temperature-shear stress curve for the same number of cycles: (a) 3 times; (b) 7 times; (c) 14 times; (d) 28 times; (e) 56 times.

The analysis of the effect of different cycle temperatures on the shear strength of concrete for the same number of cycles is shown in Fig 17 below:

As can be seen in Fig 18:

1. As the temperature increases, the shear strength of concrete tends to rise first and then fall. At 3, 7, 28 and 56 cycles of temperature and humidity, the concrete shear strength was highest at 40°C. The concrete shear strength was the highest at the circulation temperature of 80°C under the shear angle of 46° and 54° at 14 times of warm and wet cycles. Temperature and humidity cycle action 3 times, 7 times, 14 times, 28 times, 56 times, the cycle temperature of 20°C concrete shear strength of the lowest. The above phenomenon is due to the role of 20°C temperature and humidity cycle, the concrete hydration reaction is slow, the strength is slow to improve; 40°C temperature and humidity cycle, the concrete hydration reaction is faster, the strength is faster; 80°C temperature and humidity cycle, the concrete hydration reaction is rapid, releasing a lot of heat energy, so that the concrete strength is rapidly increased while generating a large number of cracks, after the dry and wet cycle of erosion, the concrete strength reduced.
2. The shear strength of concrete at shear angle 62° was the lowest at 3, 7, 14, 28 and 56 times of temperature and humidity cycling. The overall shear strength of concrete at shear angle 54° was the highest at 3 times and 56 times of temperature and humidity cycles, and the overall shear strength of concrete at shear angle 62° was the highest at 7 times, 14 times and 28 times of temperature and humidity cycles.

#### 4.2.3. Analysis of the effect of temperature and humidity cycling on the cohesion and internal friction angle of concrete

**Fig 19:**
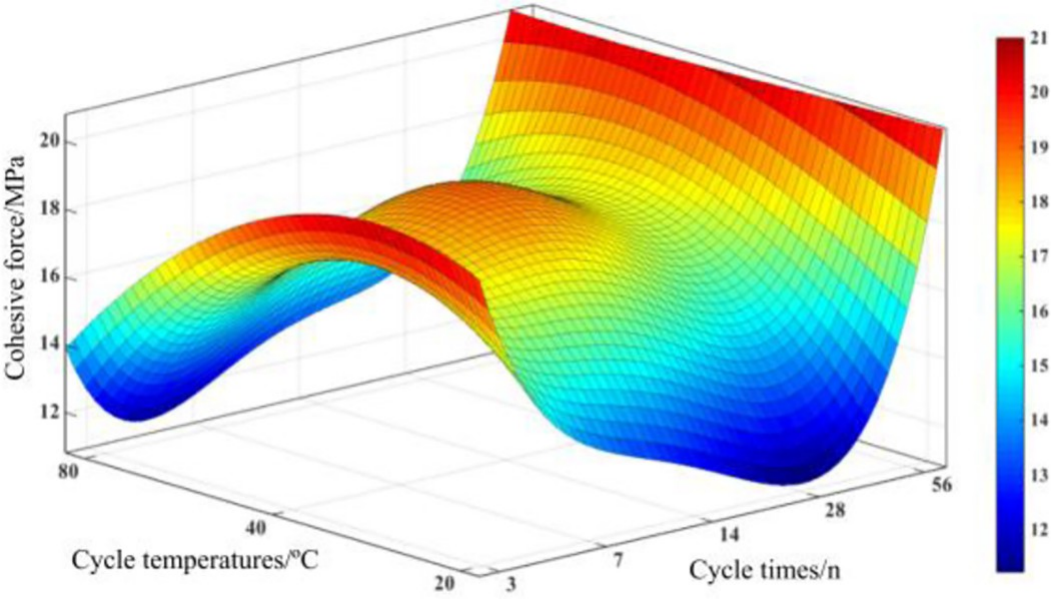
Circulation temperature-number of cycles curve.

The positive and shear stresses in concrete under the action of temperature and humidity cycles at shear angles of 46°, 54° and 62° were obtained and fitted using the shear strength formula to obtain the calculated parameters of shear strength of concrete under the action of temperature and humidity cycles, as shown in Fig 19.

**Fig 20:**
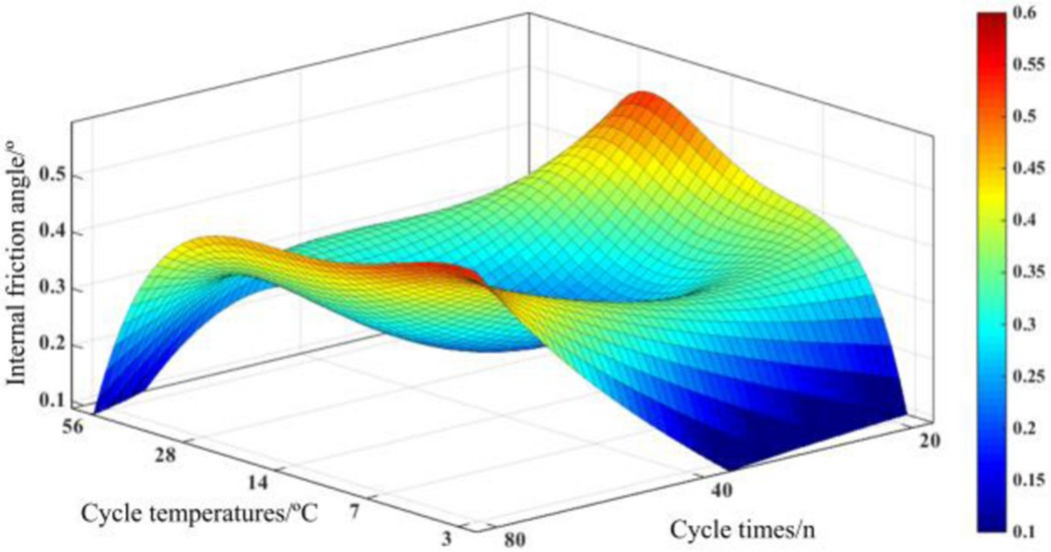
Circulation temperature-number of cycles curve.

The calculated parameters of concrete shear strength under the action of temperature and humidity cycle, and import the data of cohesion c into MATLAB software for fitting, we can find the relationship between cohesion c and cycle temperature T and cycle number n. The fitting surface is shown in Fig 20.

$$\begin{array}{l} c=40.01-43.95\times n\;-4.856\times T+19.06\times n^{2}+17.65\times n\times T+0.057\times T^{2}-4.182\times n^{3}-2.949\times n^{2}\times T\\ -4.015\times n\times T^{2}+0.082\times T^{3}+0.3853\times n^{4}-0.049\times n^{3}\times T+0.795\times n^{2}\times T^{2}-0.041\times n\times T^{3},R^{2}=0.991 \end{array}$$ (1)

c is the cohesive force, n is the number of temperature and humidity cycles, and T is the temperature of temperature and humidity cycles.

The relationship between the internal friction angle *ϕ* and the cycle temperature T and the number of cycles n can be found by fitting the data of the internal friction angle *ϕ*. The fitted surface is shown in Fig 17, and the relationship is shown in Eq (2).

$$\begin{array}{l} \phi =-2.964+4.253\times n+1.406\times T-2.115\times n^{2}-0.682\times n\times T-0.633\times T^{2}+0.549\times n^{3}-0.022\times n^{2}\times T\\ +0.011\times n\times T^{2}+0.298\times T^{3}-0.056\times n^{4}-0.038\times n^{3}\times T+0.157\times n^{2}\times T^{2}-0.057\times n\times T^{3}-0.062\times T^{4}\\ +0.015\times n^{4}\times T-0.034\times n^{3}\times T^{2}+0.012\times n^{2}\times T^{3}-0.002\times n\times T^{4}+\;0.007\times T^{5},R^{2}=0.9586 \end{array}$$ (2)

*ϕ* is the internal friction angle, n is the number of temperature and humidity cycles, and *T* is the temperature and humidity cycle temperature.

## 5. Conclusions

In this paper, the variable angle shear test under different loading rates and temperature and humidity cycles is carried out for heat-insulating shotcrete, and it is analysed that when the same shear angle is used, the peak shear stress increases and then decreases with the shear rate, and with the increase of the angle, the effect of the shear rate on the peak shear stress increases. Variable angle shear test under the action of temperature and humidity cycle, 40°C temperature and humidity cycle, with the increase in the number of cycles, the concrete strength was first increased and then decreased and then continued to increase the trend. When the temperature and humidity cycle 7 times, the concrete reaches the peak shear strength, and the highest shear strength increase under the shear angle 46°; the same number of cycles, with the increase of temperature, the shear strength of insulated shotcrete firstly rises and then decreases. Finally, the empirical equations between the two parameters of cohesion c and angle of internal friction ϕ of insulated shotcrete and the number of temperature-wet cycles n and temperature-wet cycle temperature T were fitted by using MATLAB software, respectively, which can provide a theoretical basis for concrete in engineering when it is affected by the environment of temperature-wet cycles.

## Supporting information

S1 File (PDF)
