# Supplementary material for: Variable angle of insulated shotcrete under different loading rates and temperature and humidity cycles- shear test and analysis
Source: PLoS One. 2024 Apr 18;19(4):e0297381. doi: 10.1371/journal.pone.0297381 (PMC11025942; doi:10.1371/journal.pone.0297381)
Supplement: S1 File — (PDF) [file pone.0297381.s001.pdf]

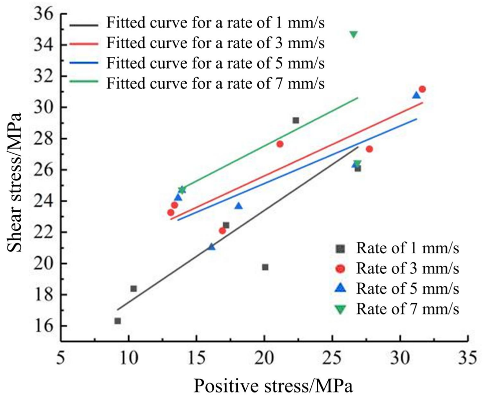

Figure 13. Different rates vs. peak shear stress curves.

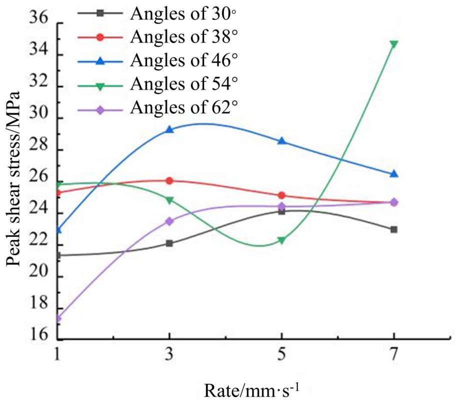

Figure 14. Specimen shear stress curves vs. positive stress curve.

Table1 Basic parameters of variable angle shearing at different rates of shotcrete

Sample Number	Loading Rate/mm·s -1	Shear Angle/°	Peak Load/kN	Normal Stress/MPa	Shearing Stress/MPa	Cohesive Force/MPa	Internal Friction Angle/°
A1	1	30	111.49	39.18	21.34	\	\
A2	3	30	115.48	40.58	22.1	\	\
A3	5	30	126.07	41.8	24.12	\	\
A4	7	30	120.04	41.18	22.97	\	\
B1	1	38	106.07	34.09	25.29	\	\
B2	3	38	109.27	35.11	26.05	\	\
B3	5	38	105.41	33.88	25.13	\	\
B4	7	38	103.53	33.27	24.68	\	\
C1	1	46	81.64	23.48	22.92	11.62	30.49
C2	3	46	104.17	29.69	29.25	17.55	21.94

C3	5	46	101.61	28.96	28.53	17.75	20.23
C4	7	46	94.21	26.86	26.45	18.55	24.17
D1	1	54	81.23	19.75	25.81	11.62	30.49
D2	3	54	78.29	19.04	24.87	17.55	21.94
D3	5	54	70.31	17.10	22.34	17.75	20.23
D4	7	54	109.26	26.57	34.72	18.55	24.17
E1	1	62	49.77	9.79	17.35	11.62	30.49
E2	3	62	67.43	13.26	23.5	17.55	21.94
E3	5	62	70.13	13.79	24.44	17.75	20.23
E4	7	62	70.91	13.94	24.71	18.55	24.17

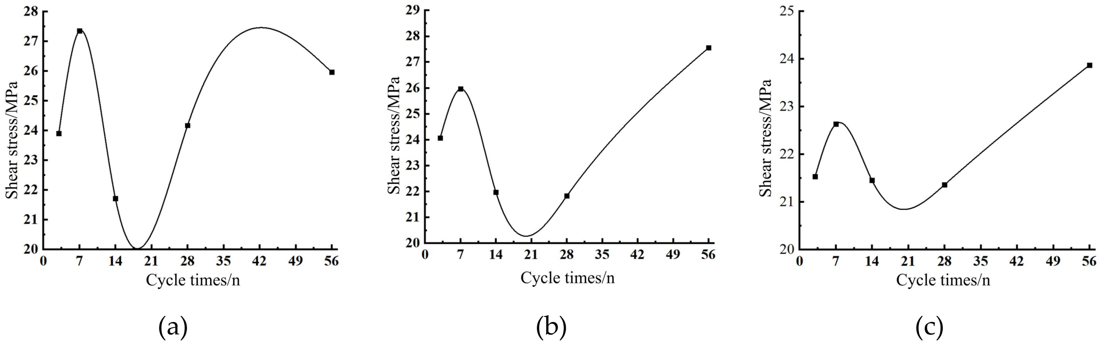

Figure 17. Different shear angle shear stress-cycle number relationship curve: (a) Shear angle 46°; (b) Shear angle 54°; (c) Shear angle 62°.

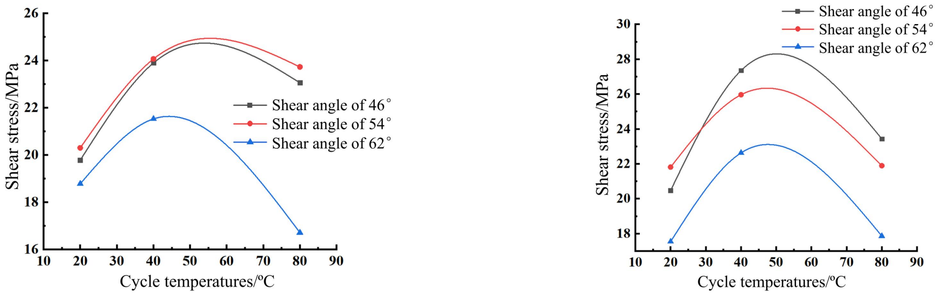

(a) (b)

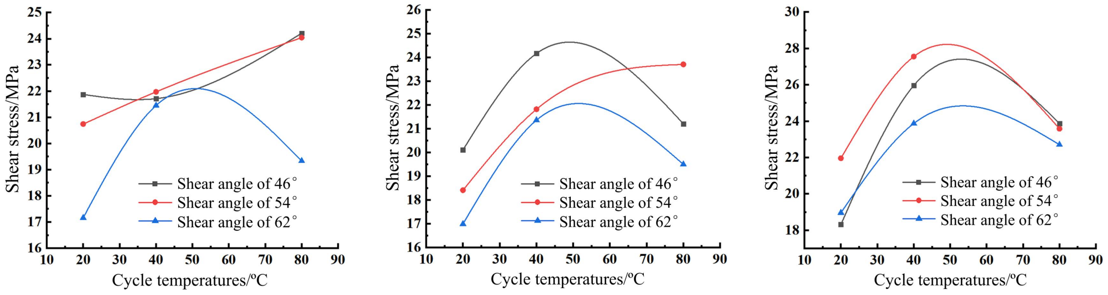

(c) (d) (e)

Figure 18. Cyclic temperature-shear stress curve for the same number of cycles: (a) 3 times;(b) 7 times;(c) 14 times;(d) 28 times;(e) 56 times.

Table2 Basic parameters humidity cycle test

Number of Cycles	Shear Angle/°	Peak Load /kN	Normal Stress/MPa	Shearing Stress/MPa	Mean Value of Normal Stress/MPa	Average Shear Stress/MPa
3	46	67.90	19.36	19.07	20.08	19.78
		77.10	21.98	21.65	20.08	19.78
		66.37	18.92	18.63	20.08	19.78
	54	61.72	15.01	19.61	15.54	20.30
		61.09	14.86	19.41	15.54	20.30
		68.90	16.76	21.89	15.54	20.30
	62	58.03	11.21	20.22	10.59	18.78
		49.72	9.78	17.34	10.59	18.78
		53.88	10.79	18.80	10.59	18.78
7	46	68.05	19.40	19.11	20.78	20.47
	46	69.12	19.70	19.41	20.78	20.47
	46	81.52	23.24	22.89	20.78	20.47
	54	64.26	15.63	20.42	16.69	21.81
	54	71.20	17.32	22.62	16.69	21.81
	54	70.46	17.14	22.39	16.69	21.81
	62	52.02	10.23	18.13	9.89	17.54
	62	48.63	9.56	16.95	9.89	17.54
	62	50.33	9.89	17.54	9.89	17.54

14	46	80.33	22.90	22.56	22.21	21.87
	46	75.47	21.51	21.19	22.21	21.87
	46	77.90	22.21	21.87	22.21	21.87
	54	69.99	17.02	22.24	15.87	20.74
	54	62.25	15.14	19.78	15.87	20.74
	54	63.56	15.46	20.19	15.87	20.74
	62	47.05	9.25	16.40	9.68	17.16
	62	46.50	9.14	16.20	9.68	17.16
	62	54.20	10.66	18.89	9.68	17.16
28	46	84.22	24.01	23.65	20.42	20.11
	46	73.61	20.98	20.67	20.42	20.11
	46	57.03	16.26	16.01	20.42	20.11
	54	63.39	15.42	20.14	14.09	18.41
	54	46.06	11.20	14.64	14.09	18.41
	54	65.43	15.91	20.79	14.09	18.41
	62	48.37	9.51	16.86	9.58	16.99
	62	50.35	9.90	17.55	9.58	16.99
	62	47.52	9.34	16.56	9.58	16.99
56	46	65.16	18.57	18.30	18.59	18.31
	46	65.36	18.63	18.35	18.59	18.31
	46	65.12	18.56	18.29	18.59	18.31
	54	66.14	16.09	21.01	16.81	21.96
	54	66..65	16.21	21.18	16.81	21.96
	54	74.55	18.13	23.69	16.81	21.96
	62	52.26	10.28	18.21	10.70	18.96
	62	50.57	9.94	17.62	10.70	18.96
	62	60.36	11.87	21.03	10.70	18.96
